# Scale-up of the production of highly reactive biogenic magnetite nanoparticles using *Geobacter sulfurreducens*

**DOI:** 10.1098/rsif.2015.0240

**Published:** 2015-06-06

**Authors:** J. M. Byrne, H. Muhamadali, V. S. Coker, J. Cooper, J. R. Lloyd

**Affiliations:** 1 School of Earth, Atmospheric and Environmental Sciences, and Williamson Research Centre for Molecular Environmental Science, University of Manchester, Manchester M13 9PL, UK; 2 Geomicrobiology, Center for Applied Geoscience, University of Tuebingen, Sigwartstrasse 10, 72076 Tuebingen, Germany; 3 Manchester Institute of Biotechnology, University of Manchester, Manchester M1 7DN, UK; 4 The Centre for Process Innovation, CPI, Wilton Centre, Wilton, Redcar TS10 4RF, UK

**Keywords:** Fe(III) reduction, remediation, bioreactor, nanotechnology, geobiology

## Abstract

Although there are numerous examples of large-scale commercial microbial synthesis routes for organic bioproducts, few studies have addressed the obvious potential for microbial systems to produce inorganic functional biomaterials at scale. Here we address this by focusing on the production of nanoscale biomagnetite particles by the Fe(III)-reducing bacterium *Geobacter sulfurreducens*, which was scaled up successfully from laboratory- to pilot plant-scale production, while maintaining the surface reactivity and magnetic properties which make this material well suited to commercial exploitation. At the largest scale tested, the bacterium was grown in a 50 l bioreactor, harvested and then inoculated into a buffer solution containing Fe(III)-oxyhydroxide and an electron donor and mediator, which promoted the formation of magnetite in under 24 h. This procedure was capable of producing up to 120 g of biomagnetite. The particle size distribution was maintained between 10 and 15 nm during scale-up of this second step from 10 ml to 10 l, with conserved magnetic properties and surface reactivity; the latter demonstrated by the reduction of Cr(VI). The process presented provides an environmentally benign route to magnetite production and serves as an alternative to harsher synthetic techniques, with the clear potential to be used to produce kilogram to tonne quantities.

## 1. Introduction

Several studies have recently highlighted the potential uses of magnetite (Fe_3_O_4_) magnetic nanoparticles for applications including targeted cancer therapies, magnetic data storage or the remediation of contaminated water and land [1–4]. Several methods of manufacturing magnetite nanoparticles currently exist; however, these often require expensive and environmentally damaging approaches [5,6]. Biological processes catalysed by dissimilatory iron-reducing bacteria, such as subsurface species of the genera *Geobacter* and *Shewanella*, offer an alternative method for the production of nanoscale ‘biomagnetite’ at ambient temperature and pressure [7–9]. These organisms conserve energy through the oxidation of organic matter as an electron donor, coupled with the reduction of poorly crystalline Fe(III) minerals (e.g. ferrihydrite) which serve as electron acceptors [10,11], with the process accelerated by the presence of electron shuttles such as humics, riboflavin or anthraquinone-2,6-disulfonate [12]. Soluble Fe(II) released by the reduction of Fe(III) results in the formation of new mineral phases such as magnetite under optimized conditions [13], although goethite, haematite, siderite or vivianite can also precipitate depending upon the formation conditions including pH and temperature [13–15]. While biomagnetite particles show considerable commercial potential, there are few studies on the scale-up of Fe(III) reduction by mesophilic subsurface Fe(III)-reducing bacteria. However, recent work has demonstrated the synthesis of biomagnetite at the 30 l scale using the thermophilic bacterium *Thermoanaerobacter* sp. TOR-39 (at 65°C) [16].

The purpose of the work described here is to demonstrate the successful scale-up of biomagnetite production at mesophilic temperatures (30°C), via reduction of ferrihydrite over relatively short time scales (less than 24 h after inoculation), while maintaining optimal surface area and reactivity of the bionanoparticles for the remediation of a model toxic metal contaminant (Cr(VI)) [17,18]. The scale-up processes presented here were separated into distinct stages: (i) optimization of growth medium to enhance biomass concentrations while maintaining high Fe(III)-reducing activity, (ii) upscaling growth of *Geobacter sulfurreducens* from 100 ml cultures to a 50 l pilot-scale bioreactor, (iii) comparison of the biotransformation of Fe(III)-oxyhydroxide with magnetite using biomass from the 50 l bioreactor in a discrete mineral biotransformation step at scales between 10 ml and 10 l, and (iv) detailed mineralogical characterization of the biomagnetite using X-ray diffraction (XRD), transmission electron microscopy (TEM), X-ray absorption (XAS) and X-ray magnetic circular dichroism (XMCD), while surface reactivity was tested by quantifying Cr(VI) reduction. Many studies have focused on the scalable biotechnological production of organic molecules including recombinant proteins as well as fine and bulk chemicals. This work addresses an important gap in the published literature on the scalable bacterial production of inorganic functional nanomaterials, widening the range of commercial products that can be potentially synthesized by biotechnological routes.

## 2. Experimental methods

### 2.1. Starting materials

Fe(III)-oxyhydroxide (commonly referred to as ferrihydrite) was precipitated by the reaction of FeCl_3_ (0.66 M) with NaOH (10 N) until pH 7 [19,20]. The material was centrifuged (17 000*g*; 20 min) and washed in d.H_2_O to remove chloride ions, with the washing repeated six times. Fe total concentration of the starting material was determined by ferrozine assay [21] after reaction with 6 M hydroxylamine hydrochloric acid.

*Geobacter sulfurreducens* cultures were grown in the dark in anoxic conditions (N_2_ : CO_2_ 80 : 20 headspace unless otherwise specified) and incubated at 30°C in a modified freshwater medium [22] containing trace elements, vitamins, electron donor sodium acetate (between 25 and 50 mM) and electron acceptor fumaric acid (between 40 and 150 mM). Full details are included in electronic supplementary material, table S2.

### 2.2. Optimization experiments

Small-scale (100 ml) experiments were prepared containing growth medium (90 ml in 100 ml serum bottle) with electron donor and acceptor concentration ratios varied between 25 : 40, 50 : 80, 25 : 100, 50 : 100, 25 : 150, 50 : 150 (donor mM : acceptor mM) with each ratio tested in triplicate. A 10% inoculum of a late log-phase *G. sulfurreducens* culture was added (grown on 25 : 40 growth medium) and incubated in the dark at 30°C without agitation. Optical density was measured at 600 nm (OD_600_) using an M501 single beam scanning UV/visible spectrophotometer at regular intervals.

Each replicate was harvested after 27 h (late log-phase of 50 : 80 medium) by centrifugation (4920*g*; 4°C; 20 min) and washed twice in NaHCO_3_ buffer (30 mM; pH 7) under a N_2_ : CO_2_ (80 : 20) gas line and finally re-suspended in NaHCO_3_ (30 mM). The optical density (OD_600_) of each suspension was measured at 600 nm, with slurries diluted with NaHCO_3_ (30 mM) buffer to achieve OD_600_ of 0.4 in 10 ml volume (0.2 ml cell suspension, 9.8 ml d.H_2_O). OD_600_ values were converted into biomass concentration corresponding to protein concentration in g l^−1^ by reference to a standard curve prepared with bovine serum albumin [23]. The dry weight biomass (g l^−1^) is determined as 55% of the total protein content [24].

Fe(III)-oxyhydroxide bioreduction experiments were prepared in volumes of 10 ml, containing electron donor (sodium acetate, 20 mM), electron acceptor (Fe(III)-oxyhydroxide, 50 mmol l^−1^), NaHCO_3_ buffer (30 mM) and electron shuttle (riboflavin, 10 μM). Fe(III) bioreduction medium was prepared under an N_2_ : CO_2_ (80 : 20) gas line to ensure anoxic conditions, and autoclaved (121°C; 20 min) in a Prestige Medical autoclave. Fe(III)-oxyhydroxide containing media were inoculated with *G. sulfurreducens* (0.073 g l^−1^ dry weight biomass) in triplicate and incubated in the dark at 30°C without agitation. Fe(II)_(aq)_ production rate was measured using the ferrozine assay [21]. The bottles were homogenized by vigorous shaking and then 0.1 ml of sample was extracted from each bottle using a sterile syringe. The extracted sample was added to a universal tube, into which 4.9 ml HCl (0.5 M) was added to acidify the sample. This was left for 1 h; 0.05 ml of this was added to 2.45 ml ferrozine solution and measured in cuvettes using an M501 single beam scanning UV/visible spectrophotometer at 562 nm.

For intermediate bioreactor experiments, an Applikon 7 l dished bottom bioreactor vessel was filled with 4.5 l growth medium (50 mM acetate, 80 mM fumaric acid; pH = 7) and sterilized at 121°C for 20 min in a Boxer 300/150 LR autoclave. The medium was purged with N_2_ gas for 30 min to make the system anoxic then all input and output valves were sealed. The pH was adjusted to pH = 7 through the use of several approaches including CO_2_, HCl (2 M), NaOH (2 M) or MOPS (3-(*N*-morpholino)propanesulfonic acid; 1 M). The medium was inoculated with 10% late log-phase culture of *G. sulfurreducens* and incubated at 30°C. Redox (mV) and pH were observed *in situ* with permanent probes (Mettler Toledo Combination redox probe Pt4805-SC-DPAS-K8S/325 and AppliSens Z001032551 pH probe). Biomass was extracted via a sample point and measured as OD_600_ at regular intervals. Impellor speed was varied from 0 r.p.m. to 100 r.p.m. over different experiments.

### 2.3. Pilot plant-scale production of bacterial cultures and mineral transformation

A Sartorius 10 l seed bioreactor was prepared with 4.5 l of the growth medium (50 mM acetate, 80 mM fumaric acid; pH = 7) and sterilized *in situ* at 121°C for 30 min. Following sterilization, the medium and vessel were purged with N_2_ gas for 30 min. The pH of the starting medium was adjusted to neutral using 1 M MOPS. The 50 l bioreactor was prepared with 45 l growth medium and sterilized at 121°C for 30 min. Minor changes to the pH occurred during the autoclaving process, with the medium then purged with N_2_ gas and adjusted to neutral pH using 1 M MOPS.

The 10 l seed fermenter was inoculated with 10% late log-phase culture of *G. sulfurreducens* and incubated at 30°C, and gentle stir speed (100 r.p.m.). There was no pH control after inoculation. After 43 h growth in the seed fermenter, 5 l *G. sulfurreducens* was transferred to the 50 l reactor (10% inoculum). Again, pH was not controlled and the temperature was kept constant at 30°C, stir speed 100 r.p.m.

Fe(III) bioreduction media were prepared in d.H_2_O containing electron donor (sodium acetate, 20 mM), electron acceptor (Fe(III)-oxyhydroxide, 33 mmol l^−1^) and NaHCO_3_ buffer (30 mM) in volumes of 2 × 10 l, 3 × 1 l, 10 × 100 ml and 10 × 10 ml. Fe(III) bioreduction media (pH ≈ 9) were then autoclaved (121°C; 20 min); 10 µM riboflavin was added as an electron shuttle to each bottle and purged with N_2_ to make anoxic, with pH adjusted (pH = 7) using 1 M MOPS. After a 48 h biomass production phase, the entire contents of the 50 l fermentation vessel was centrifuged in a Thermo Scientific Sorval RC 3BP+ centrifuge (7000*g*, 4°C; 20 min) to concentrate biomass. The bacterial culture was harvested in batches of 6 × 1 l. Following the removal of supernatant, cell pellets were re-suspended in NaHCO_3_ solution (30 mM; pH 7) and centrifuged again, with this washing repeated twice. The final cell pellet was re-suspended in approximately 900 ml of NaHCO_3_ (30 mM) solution to form the bacterial cell slurry. The OD_600_ of the slurry was measured using a Jenway 6305 spectrophotometer. Inocula of 320 ml, 32 ml, 3.2 ml and 0.32 ml *G. sulfurreducens* concentrated cell suspensions were added to each 10 l, 1 l, 100 ml and 10 ml bottle, respectively, and incubated in the dark at 30°C.

Fe(II)_(aq)_ concentrations in Fe(III)-bioreduction experiments were measured using ferrozine assay [21] at intervals of 0 h, 4 h, 24 h and 72 h. Measurements were repeated in triplicate for the 10 ml, 100 ml and 1 l Fe(III)-bioreduction experiments. Triplicate measurements of the 10 l volume were achieved from a single magnetite production bottle, with vigorous shaking performed between taking each sample.

Mineral phases present in post-reduction products were identified using powder XRD, measured with a Bruker D8 Advance instrument with Cu *K_*α*_*_1_ source. Spectra were collected over a 2*θ* range of 5–70° with step size of 0.02°. Average crystallite particle size of magnetite was determined from the (220) and (311) reflections using the Scherrer equation [25].

Imaging of the post-reduction products was performed using TEM carried out using a Phillips/FEI Technai electron microscope equipped with a field emission gun (FEG) and Gatan image filter (GIF). Images are presented in bright-field with an operating beam voltage of 300 keV.

XAS on the Fe *L*_2,3_-edge of the samples were measured at the Advanced Light Source, Lawrence Berkeley National Laboratory, Berkeley, CA, USA. Data were acquired in total-electron yield mode using circularly polarized light with XAS collected with the sample in an applied magnetic field of ±0.6 T, parallel and anti-parallel to the direction of the beam. XMCD data were derived from the difference between the XAS spectra collected under both magnetic field directions. XMCD spectra are dependent on magnetization, site location and valence state (i.e. number of *d* electrons) and are used to determine the oxidation state of cations occupying different lattice sites in metal oxides [26]. Site distributions of Fe cations within the crystalline material were determined through the application of atomic multiplet calculations [27,28]. Dried samples were loaded on to carbon tape mounted onto a copper sample probe in an anoxic glove bag purged with N_2_. The sample probe was transported to the sample chamber in an airtight container and loaded in a back flow of nitrogen to minimize potential oxidation.

Mössbauer spectra were recorded with a FAST ComTek 1024-multichannel analyser system using a constant acceleration drive (room temperature, *γ*-source approximately 25 mCi ^57^Co/Rh matrix). Samples were sealed between two layers of kapton tape in an anoxic glove bag to prevent oxidation. Measurements at low temperatures were obtained using a liquid nitrogen bath cryostat. Spectra were fitted using Voigt-based fitting in Recoil (University of Ottawa) [29]. Isomer shift data were calibrated with reference to metallic Fe foil (thickness 4 mg Fe cm^−2^) recorded at room temperature.

Surface reactivity of biogenic magnetite produced was tested by quantifying the Fe(II)-mediated reduction of Cr(VI). Fe(III) bioreduction products were washed twice in d.H_2_O, using magnetic separation resuspended to final concentration of 50 mmol l^−1^ Fe (by ferrozine assay) in a 5 mM solution of K_2_CrO_4_. All bottles were mixed continuously in the dark on a roller-mixer at 33 r.p.m. (20°C). Aliquots of 0.2 ml were extracted and centrifuged at 15 000*g* for 1 min to separate solid material from solution; 50 µl aliquots of the supernatant were diluted 1 : 100 with d.H_2_O and used to measure Cr(VI)_(aq)_ using diphenyl carbazide spectrophotometric assay [30] with absorption measured at 540 nm.

## 3. Result and discussion

### 3.1. Scale-up of *Geobacter sulfurreducens* production

**Figure 1.**
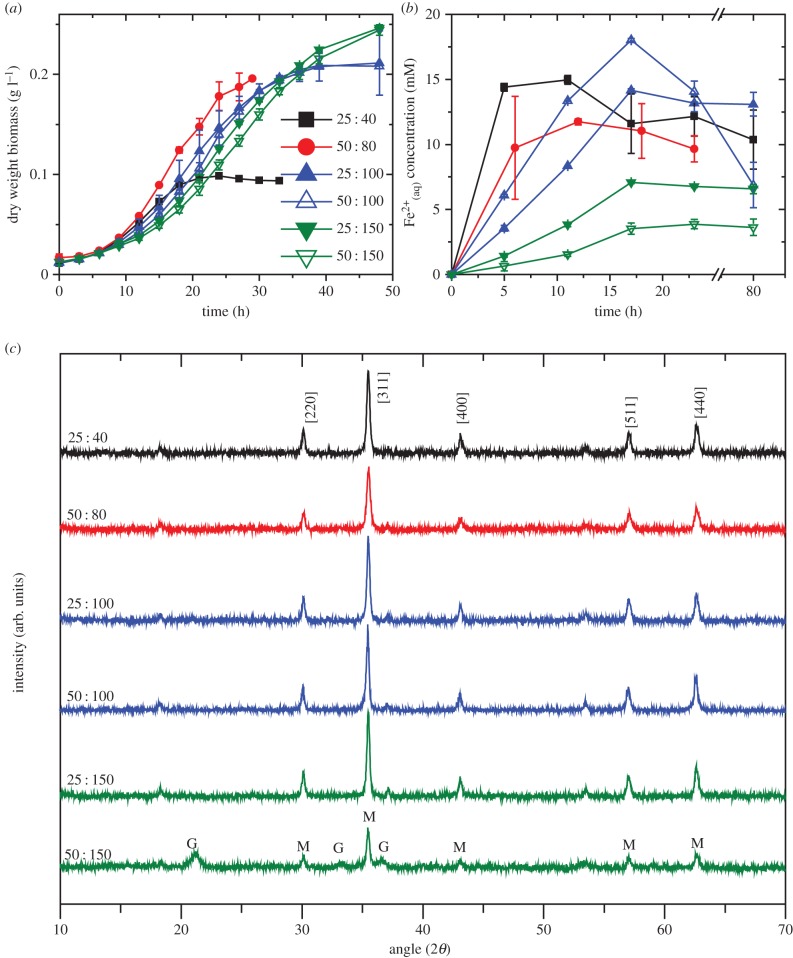
Optimization of growth medium. (*a*) Growth rate of *Geobacter sulfurreducens* with varied electron donor : acceptor ratios. (*b*) Fe(III) reduction rates of bacteria produced with varied electron donor : acceptor ratios. (*c*) XRD of the end product indicates mostly magnetite (M) formation with goethite (G) present in sample 50 mM acetate : 150 mM fumarate. (Online version in colour.)

The growth medium used to produce increased microbial biomass levels was optimized using small-scale (100 ml) incubations. Previous studies used modified fresh water medium [22], with electron donor (acetate) and electron acceptor (fumarate) concentration ratio of 25 : 40 mM, respectively, as well as trace metals, vitamins and carbonate buffer yielding biomass of approximately 0.2 g protein l^−1^ (equivalent to approximately 0.11 g biomass dry weight l^−1^ [31]). Here the electron donor : electron acceptor ratio was varied between 25 : 40, 50 : 80, 25 : 100, 50 : 100, 25 : 150 and 50 : 150 mM (figure 1). Increasing electron acceptor concentration led to an increase in total biomass production compared to the standard 25 : 40 mM (figure 1*a*), with biomass more than doubling from 0.094 g l^−1^ dry weight (25 : 40) to 0.248 g l^−1^ dry weight (25 : 150). Increasing electron donor concentration in these experiments did not affect the final biomass density, suggesting that the electron acceptor was the limiting nutrient in the standard growth medium. This is potentially related to the high electron storage capacity observed in the cytochromes of *G. sulfurreducens* [32], with higher electron acceptor concentrations increasing the redox cycling capacity of the bacteria.

Fe(III) bioreduction rates were monitored through the measurement of Fe(II)_(aq)_ released during the experiment. *Geobacter sulfurreducens* grown in 25 : 40 medium exhibited the fastest reduction rates, while samples containing 25 : 150 and 50 : 150 ratios of electron donor : acceptor showed the slowest Fe(III) reduction rates (figure 1*b*). Thus, cultures tending towards electron acceptor limitation appear to have optimal Fe(III)-reducing capacity. Doubling the concentrations of both donor and acceptor (50 mM acetate, 80 mM fumarate) maintained high Fe(III)-reducing activities, while increasing biomass yields.

The end products of Fe(III) bioreduction were examined visually for colour and magnetization using a bar magnet. While the colour of the 25 : 40 samples was dark black and strongly magnetic (consistent with magnetite), the 25 : 150 and 50 : 150 samples had a dark brown/grey colour and were less strongly attracted to the magnet. Sample 50 : 80 behaved similar to 25 : 40 samples. Further analysis using powder XRD (figure 1*c*) confirmed the presence of magnetite in all samples with goethite (α-FeOOH) observed in the 50 : 150 ratio sample, probably due to the low reduction rate observed in figure 1*b* resulting in incomplete recrystallization of ferrihydrite to magnetite [14]. The average crystallite sizes of the magnetite nanoparticles were determined to be 43 nm, 32 nm, 51 nm, 55 nm, 57 nm and 51 nm for 25 : 40, 50 : 80, 25 : 100, 50 : 100, 25 : 150 and 50 : 150, respectively.

From the results presented in figure 1, a concentration of 50 mM acetate and 80 mM fumarate was chosen for *G. sulfurreducens* biomass growth for magnetite production during scale-up work, offering a good biomass yield together with fast Fe(III) reduction rates. The benefits of choosing higher concentrations of electron donor and acceptor (at similar ratios) were not considered to be sufficient to justify the potential additional economic costs of the medium constituents, although there may be further scope for medium optimization if other limiting nutrients are identified at these higher concentrations of electron donor and acceptor.

**Figure 2.**
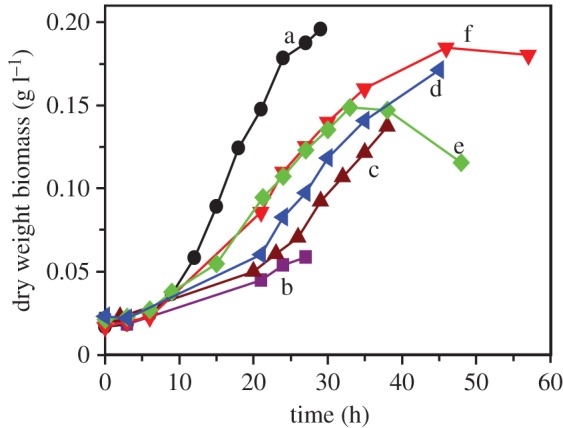
Growth of *Geobacter sulfurreducens* in a 5 l bioreactor: (*a*) 100 ml, 0 r.p.m. stirrer speed, no pH control during experiment; (*b*) 5 l, 100 r.p.m. stirrer speed, pH controlled with 2 M HCl and 2 M NaOH; (*c*) 5 l, 100 r.p.m. stirrer speed, pH controlled with 1 M MOPS (pH 4.1) and 2 M NaOH; (*d*) 5 l, 100 r.p.m. stirrer speed, no pH control during experiment; (*e*) 5 l, 0 r.p.m. stirrer speed, no pH control; (*f*) 5 l, 50 r.p.m. stirrer speed, no pH control. (Online version in colour.)

An intermediate scale bioreactor (7 l) was used for biomass production containing the defined medium with 50 mM acetate and 80 mM fumarate. The initial growth medium experiments were 100 ml bottles that were de-gassed (and pH neutralized) with an 80 : 20 N_2_ : CO_2_ gas mix before being sealed and autoclaved. Sterilization using this approach was not possible in the bioreactor, as the pressure vessel was unable to withstand high pressures without using venting ports. These venting ports permitted the backflow of oxygen into the system post autoclaving. Additionally, only N_2_ gas was available in the pilot plant for the 50 l fermenter, hence an alternative method other than gassing with 20% CO_2_ was required to lower the pH of the bicarbonate-containing growth medium to pH 7. Figure 2 shows the growth of *G. sulfurreducens* measured in the 7 l bioreactor containing 5 l medium and with a range of pH control methods (figure 2*a*).

One benefit of working with a bioreactor rather than with sealed bottles is the ability to closely control pH. When 2 M HCl and 2 M NaOH were used as acid and base respectively, to control pH (at pH 7), a relatively poor growth rate was recorded (figure 2*b*) despite the fact that only relatively minor amendments of acid and base were used during the control procedure. This was also the case when 1 M MOPS and 2 M NaOH were used to control pH (figure 2*c*). The reasons for this inhibition of growth rate during pH control with minor amendments were not clear; however, it became apparent that the carbonate buffer system already present in the modified growth medium proved sufficient to maintain a circumneutral pH. In the absence of a controlled CO_2_ headspace, 1 M MOPS was used to initially neutralize the pH of the medium following the de-gassing stage and prior to addition of the inoculum. Stirring at 100 r.p.m. had a negative impact on growth (figure 2*d*), with a slower growth rate noted than for 0 r.p.m. and 50 r.p.m. (figure 2*e*,*f*). The absence of stirring (0 r.p.m.; figure 2*e*)), however, resulted in the formation of a biofilm on the inside of the reactor vessel, which can be observed by the decrease in planktonic biomass after approximately 33 h of growth, hence a minimal amount of stirring (50 r.p.m.; figure 2*d*) was considered necessary.

### 3.2. Large-scale cultivation of *Geobacter sulfurreducens* and biomagnetite synthesis

Following on from small- (100 ml) and intermediate-scale (5 l) optimization experiments, the most suitable procedures which provided high bacterial yield and high purity of magnetite nanoparticles were transferred to a pilot-plant system for further scale-up work. Specifically, these included the use of (i) 50 : 80 acetate to fumarate concentration in the growth medium together with trace elements and vitamins, (ii) MOPS to neutralize the starting pH before inoculation, (iii) no pH control during the incubation, and (iv) an impellor rotation speed of 100 r.p.m. The procedure was divided into two stages focusing on large-scale growth of the bacterium *G. sulfurreducens* in a 50 l bioreactor, and microbial Fe(III) reduction in reaction vessel volumes ranging from 10 ml to 10 l (electronic supplementary material, figure S1).

**Figure 3.**
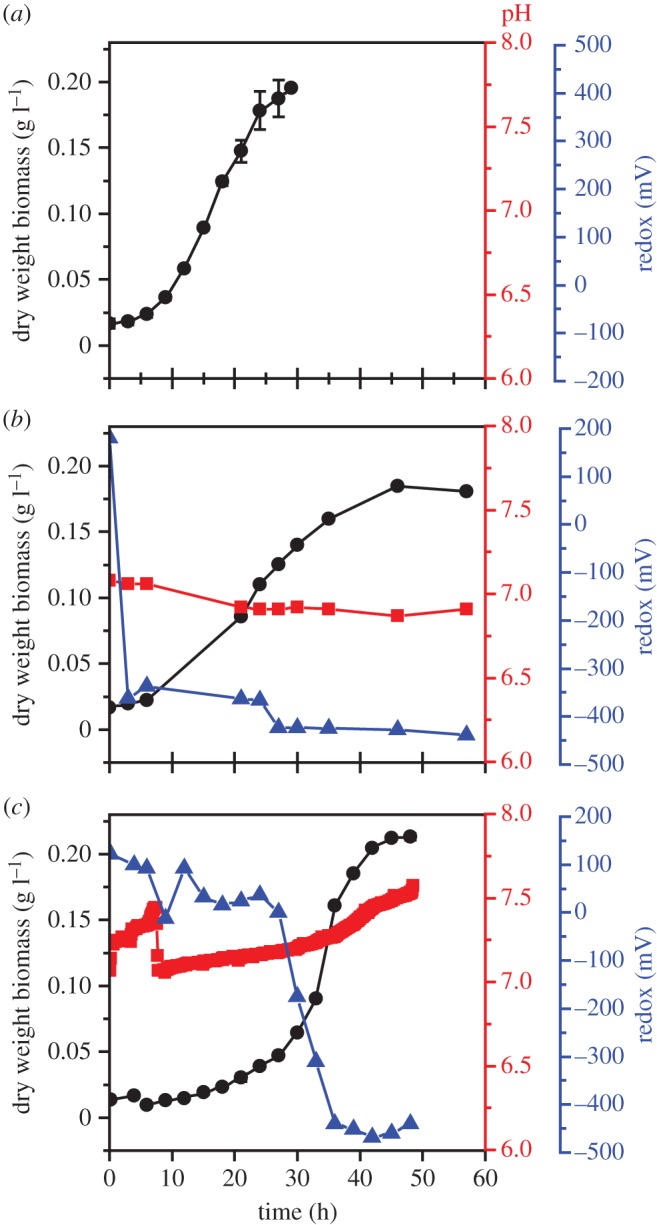
*Geobacter sulfurreducens* growth curves: (*a*) 100 ml bottles; (*b*) 5 l bioreactor; (*c*) 50 l bioreactor. Biomass measured as dry weight protein (g l^−1^), represented by (circle), pH (square) and redox (mV) (triangle); pH was not controlled during the experiments. (Online version in colour.)

Biomass density (OD_600_ converted to g l^−1^ dry weight), redox (mV) and pH of the *G. sulfurreducens* culture were monitored over 48 h in 5 l and 50 l bioreactors with results compared to the growth profile obtained in 100 ml bottles (figure 3). A 10% inoculum from a late exponential phase starter culture was used throughout. There was a significant increase in the lag phase of the cultures with increasing vessel volume, with the 100 ml culture exhibiting the shortest lag phase. The reasons for this were not clear; however, it might be due to the presence of an impellor which was not present for the 100 ml samples, but was for 5 l and 50 l bioreactors. The impellor was used to prevent the formation of a biofilm on the walls of the vessels; however, it is not known what effects the mixing of the culture medium could have on microbial growth. The doubling time was approximately 5.7 h and 3.8 h for the 100 ml and 50 l cultures, respectively. Final biomass yields were similar for all volumes: 0.198 g l^−1^, 0.187 g l^−1^ and 0.215 g l^−1^ (dry weight) for 100 ml, 5 l and 50 l production runs, respectively. The pH in 5 l and 50 l cultures remained circumneutral, with a minor decrease seen in the 5 l bioreactor and a minor increase seen in the 50 l bioreactor. Redox values in both vessels decreased as cultures grew, with a maximal drop from +180 to −362 mV and from +37 to −440 mV for 5 l and 50 l cultures, respectively.

**Figure 4.**
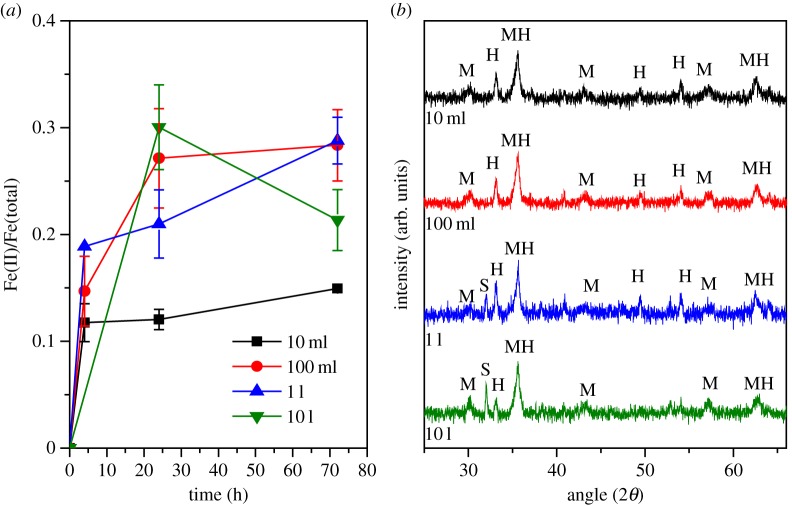
Fe(III) reduction rates and formation of iron oxide minerals. (*a*) Fe(II)/Fe(total) reduction rates. (*b*) Powder XRD indicates all samples contain magnetite (M) and haematite (H) with siderite (S) produced in 1 l and 10 l samples. (Online version in colour.)

For magnetite production, *G. sulfurreducens* were harvested from the 50 l bioreactor after 49 h of batch growth. A bacterial slurry (approx. 900 ml) was produced with final biomass of 5.8 g l^−1^ dry weight. Inocula (0.185 g l^−1^ dry weight) of the slurry were added into 33 mmol l^−1^ Fe(III)-oxyhydroxide suspensions. Based on this inoculum, 900 ml bacterial slurry harvested from the 50 l fermenter was sufficient for the inoculation of approximately 28 l of Fe(III)-oxyhydroxide biotransformation medium. To examine the effect of scale-up on the biotransformation of ferrihydrite to magnetite, this process was carried out in 10 ml, 100 ml, 1 l and 10 l bottles, each containing the same concentrations per litre of electron donor, electron acceptor, buffer and electron shuttle. Fe(III) reduction rates were monitored over 72 h (figure 4*a*). Results indicated only minor differences in the reduction rates of Fe(III) to Fe(II) at all scales. Figure 4*a* displays the rate of Fe(II) generation relative to the total Fe present at the start of the reaction. Stoichiometric magnetite (Fe_3_O_4_) has a Fe(II)/Fe(tot) ratio of 0.33 (i.e. one-third of the total Fe should be Fe(II)). Figure 4*a* shows little differences between the 100 ml, 1 l and 10 l transformations in terms of Fe(II) generation, with all three curves within error of each other; however, each fell below 0.33 Fe(II)/Fe(tot). The smallest volume vessel (10 ml) exhibited significantly less overall Fe(III) reduction, with a final value of only 0.19 Fe(II)/Fe(tot). All samples were dark black with magnetic characteristics, consistent with magnetite, after only 4 h of incubation. The reasons for the lower than expected final concentration of Fe(II) could potentially be (i) that the biomagnetite is partially oxidized in comparison with stoichiometric magnetite, (ii) due to the limitation of the assay, which used a 0.5 M HCl digestion step that may not fully dissolve all of the Fe present in the magnetite structure [33], or (iii) due to the presence of additional mineral phases which are discussed below in more detail. The density of the final product was 4.3 g l^−1^ (±1.6) dry weight, which, assuming that up to 28 l Fe(III)-oxyhydroxide suspension can be inoculated, corresponds to a final yield of approximately 120 g magnetite from the total amount of *G. sulfurreducens* cells harvested from the 50 l bioreactor.

Powder XRD indicated the presence of magnetite (Fe_3_O_4_) and haematite (Fe_2_O_3_) in all samples, with siderite (FeCO_3_) observed in the 1 l and 10 l vessel samples (figure 4*b*). ^57^Fe Mössbauer spectroscopy indicated a relative abundance of 76% magnetite, 0% siderite and 24% haematite in the 10 ml experiment, and 72% magnetite, 6% siderite, 23% haematite in the 10 l experiment (electronic supplementary material, table S1 and figure S3). The presence of haematite may explain why the data in figure 4*a* did not reach the optimal ratio of 0.33 Fe(II)/Fe(tot) for magnetite, as this mineral structure contains Fe(III) which is not bioavailable to *G. sulfurreducens* for enzymatic reduction [34]. The haematite detected in all of the samples was an artefact resulting from the autoclaving procedure, with the effect exacerbated by autoclaving at pH 9 before de-gassing with N_2_ [13].

The average crystallite particle size of the magnetite was calculated from XRD; however, owing to presence of three mineral phases with overlapping reflections (i.e. haematite (110) at 35.6° (2*θ*) and magnetite (311) at 35.4° (2*θ*)), it is difficult to accurately determine the particle size using the most intense magnetite reflection. Instead the next most prominent (220) peak was used. Results indicated minor differences in the particle sizes of the magnetite produced with 12.3 nm, 10.6 nm, 14.4 nm and 15.0 nm diameter particles present in the 10 ml, 100 ml, 1 l and 10 l biomagnetite production steps, respectively. From previous work, it is known that the size of the nanoparticles is related to the concentration of biomass introduced at the start of the biotransformation, with higher loadings of bacteria leading to the production of smaller nanoparticles [35]. This is considered to be a rate-dependent effect, with higher amounts of Fe(II) production leading to an increase in the probability of formation of nucleation sites [36–38]. The size of the particles produced follows closely the results obtained for magnetite nanoparticles produced with similar starting biomass values (13.7 nm from an inoculation of 0.209 g l^−1^ dry weight) [35].

TEM and electron diffraction were performed on materials produced in 10 ml and 10 l volume transformations (electronic supplementary material, figure S2). The results of TEM imaging were consistent with the XRD analyses, with haematite phases clearly observable at the same time as magnetite (electronic supplementary material, figure S2*a*(iii),*b*(iii)), confirmed by selected area electron diffraction analyses (electronic supplementary material, figure S2*a*(ii),*b*(ii)). The sizes of the magnetite nanoparticles were 16.9 ± 2.6 nm and 12.8 ± 2.0 nm for samples from the 10 ml and 10 l magnetite synthesis step, respectively, in close agreement with XRD average crystallite size data (figure 4*b*). The size of the haematite produced was approximately 75 nm and 74 nm for 10 ml and 10 l, respectively. Electronic supplementary material, figure S2*b*(iii) contains three spherical particles with an average size of 208 ± 24 nm, which match the morphology that has been observed previously for iron carbonate biominerals, namely siderite, formed by *Shewanella* sp., i.e. disc-like [39]. Large particles of this type were not observed in the images obtained from the 10 ml sample.

The presence of siderite (FeCO_3_) has previously been reported when magnetite was produced by a high density culture of *G. sulfurreducens* [35], attributed to excess Fe(II) which reacted with the HCO_3_^−^ in solution to produce the Fe(II)-carbonate. Siderite was only observed in samples from the 1 l and 10 l biomagnetite production experiments, and not noted in the 10 ml or 100 ml experiments.

**Figure 5.**
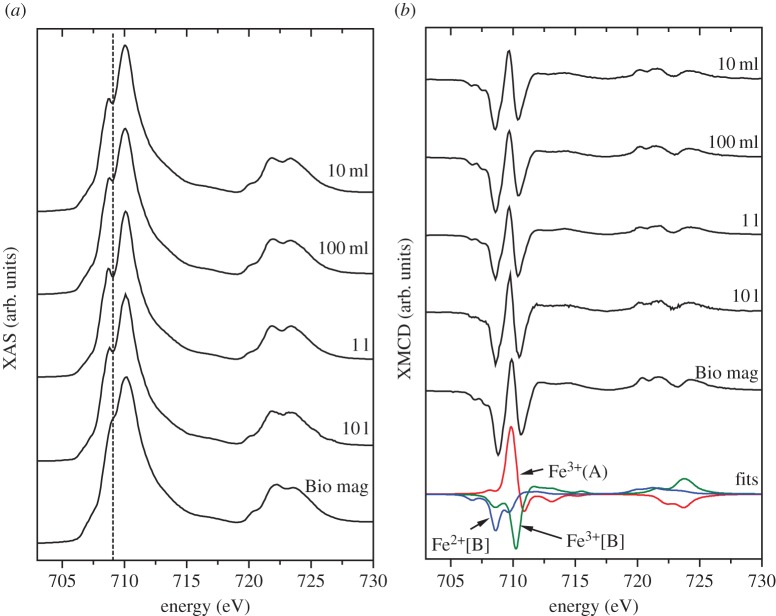
(*a*) X-ray absorption spectra (XAS) of samples obtained through Fe(III) reduction in volumes of 10 ml, 100 ml, 1 l and 10 l. ‘Bio mag’ corresponds to sample of biogenic magnetite [35] with close to stoichiometric cation distribution. (*b*) X-ray magnetic circular dichroism (XMCD) with peaks corresponding to Fe^2+^[B], Fe^3+^(A) and Fe^3+^[B] as highlighted by fits in blue, red and green, respectively. (Online version in colour.)

Synchrotron radiation techniques, XAS and XMCD were used to determine the distribution of iron in the biomagnetite (figure 5). Magnetite has a cubic spinel structure with Fe^2+^ and Fe^3+^ cations arranged in tetrahedral (A) and octahedral [B] coordination according to (Fe^3+^)_A_[Fe^2+^Fe^3+^]_B_O_4_^2−^. Anti-ferromagnetic coupling between the two lattice sites effectively cancels out the magnetic moments of Fe^3+^ cations, resulting in net magnetization owing to Fe^2+^. The XAS of the Fe *L*_2,3_ edges (formed from the average of±0.6 T spectra) (figure 5*a*) showed little differences between each sample despite the presence of siderite in 1 l and 10 l samples (haematite was present in all samples), indicating that it is only a minor component. Spectra were compared with a sample of biogenic magnetite from small-scale culture, reported previously [35], designated ‘Bio mag’ and known to have a close to stoichiometric cation distribution (table 1) with a slight excess of octahedral Fe^2+^[B], (Fe^2+^/Fe^3+^ 0.52). This is characterized by a smooth shoulder on the low energy side of the peak intensity (dashed line). The shoulder is more pronounced in the 50 l fermenter derived samples, which is indicative of a less reduced sample (i.e. less Fe^2+^[B] in relation to other Fe cations). However, it is more likely to be due to the presence of the Fe(III) oxide haematite which is not present in the reference ‘Bio mag’ sample [35].

**Table 1.** Fe cation distribution within the magnetite nanoparticles, with values determined through fitting of XMCD spectra.

sample	diameter (nm)	Fe^2+^[B]	Fe^3+^(A)	Fe^3+^[B]	Fe^2+^/Fe^3+^
10 ml	12.3	0.99	0.96	1.06	0.49
100 ml	10.6	1.01	0.95	1.05	0.51
1 l	14.4	0.78	0.99	1.24	0.35
10 l	15.0	0.89	1.01	1.11	0.42
Bio mag	13.7	1.03	0.96	1.02	0.52

Further crystallographic information was obtained using XMCD. Haematite and siderite are anti-ferromagnetic [40] and do not contribute to the XMCD spectra. This allows the technique to probe the stoichiometry of only the magnetite. The intensity of the spectra provides a relative measure of magnetization of the nanoparticles assuming the average of the two associated XAS spectra are normalized to 1. Using this approach it is clear that there are no obvious differences between the magnetization of the magnetite produced at different scales. Further quantitative analysis of the stoichiometry and relative Fe cation site occupancy of the magnetic was obtained through spectral fitting with XMCD peaks corresponding to octahedral Fe^2+^[B] (first negative peak), tetrahedral Fe^3+^(A) (positive peak) and octahedral Fe^3+^[B] (second negative peak) (table 1). Stoichiometric magnetite (Fe^3+^)_A_[Fe^2+^Fe^3+^]_B_O_4_^2−^ has Fe^2+^/Fe^3+^ ratio of 0.5. The materials from 10 ml and 100 ml experiments displayed almost exact stoichiometric occupancy (close to the reference ‘Bio mag’), while 1 l and 10 l derived materials appeared to have less than stoichiometric Fe^2+^[B] balanced by a higher Fe^3+^[B] (table 1). These results suggest the materials produced at 1 l and 10 l are slightly oxidized in comparison with those produced at smaller volumes.

**Figure 6.**
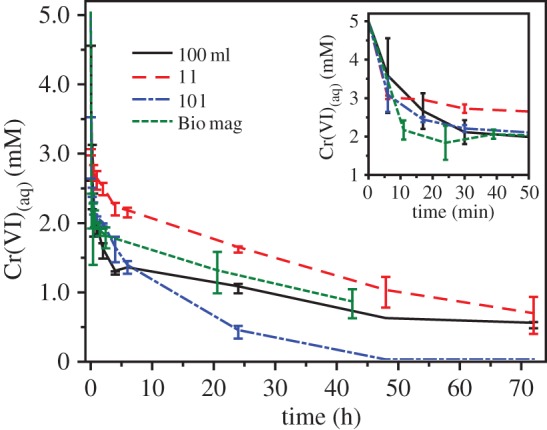
Biomagnetite surface reactivity with Cr(VI). Most of the removal of aqueous Cr(VI) occurred within the first 30 min of reaction (inset graph) and it is clear that the reduction rate of ‘Bio mag’ is within error of the 10 l sample. (Online version in colour.)

Surface reactivities of the materials were tested against chromate (Cr(VI)), demonstrating their effectiveness towards toxic metal remediation through Fe(II)-mediated reduction/sequestration. One particular focus was the effect of producing the magnetite nanoparticles from *G. sulfurreducens* grown at large volume compared with small volumes. Materials prepared in the Fe(III)-bioreduction vessels at 100 ml, 1 l and 10 l volumes were re-suspended in 10 ml serum bottles with total Fe concentration normalized to 50 mmol l^−1^. Each was reacted with potassium chromate (K_2_CrO_4_; 5 mM) with Cr(VI) concentration monitored over 72 h. The effectiveness of these magnetic nanoparticles for the removal of Cr(VI) was compared with ‘Bio mag’ reference sample. Results (figure 6) indicated that after only 6 min 30%, 40%, 39% and 48% of the Cr(VI) was removed from solution for samples obtained at the 100 ml, 1 l, 10 l scales and Bio mag, respectively. After 6 h, the 10 l sample continued to reduce/sequester Cr, reaching compete Cr(VI) removal at 48 h. Samples from the smaller volume experiments (100 ml and 1 l) did not go to completion, having removed 90% and 86% Cr(VI) from solution, respectively, after 72 h. The comparison between samples indicates very little difference between the reactivities of the magnetite nanoparticles irrespective of production volume (100 ml, 1 l or 10 l) or through the use of Fe(III)-reducing bacteria produced at differing volumetric scales (1 l for ‘Bio mag’ rather than 50 l for the other samples). Previous studies have indicated that the reaction of biogenically derived magnetite with Cr(VI) leads not only to the complete reduction of Cr(VI) to Cr(III), but also to the incorporation of the Cr(III) into the mineral structure in place of octahedrally bound Fe(II) [17,18,41]. Cutting *et al.* also demonstrated the superior reducing capacity of biogenically derived magnetite, compared with synthetic magnetite. In addition, the experiment indicates that despite the presence of additional mineral phases such as haematite and siderite, biogenic magnetic nanoparticles produced at large scale from a batch fermentation of *G. sulfurreducens* are highly capable of the remediation of Cr(VI).

## 4. Conclusion

This study has shown that biogenic magnetite nanoparticles can be produced using large-scale batch cultures, with only minor differences observable between the final products of small- and large-scale iron transformations. The material produced was tested and shown to be suitable for remediation of chromate contaminated waste waters, with little impact of scaling on the material's ability to remove Cr(VI) from solution. It should be noted that previous studies have shown that microbiologically synthesized magnetite nanoparticles offer superior performance to synthetic analogues for this and other applications [34]. Many of the approaches which have been applied here could in practice be applied to industrial systems which are several orders of magnitude larger. However, some careful measures should be taken including:

(1) For *G. sulfurreducens,* the optimum electron donor to acceptor ratio is 50 mM acetate to 80 mM fumarate in order to yield high cell density and rapid Fe(III)-reducing capacity.
(2) Design of larger-scale bioreactor configuration may require optimization. For example, it might be possible to control the lag phase through modifications to the mixing regime, with the use of the fermenter impeller implicated in prolonging the growth phase during scale-up.
(3) Fe(III)-oxyhydroxide (i.e. ferrihydrite) starting materials should not be autoclaved to minimize potential haematite formation. Microbial contamination will not be an issue here as the concentrations of *G. sulfurreducens* used are so high.
(4) The formation of siderite can be prevented by choosing an alternative buffer system to carbonate in the mineral transformation stage.
(5) The large-scale procurement of suitable substrates, including the bioavailable Fe(III) mineral phase for efficient microbial reduction to magnetite, also requires attention. This is needed for accurate cost–benefit calculations that must be made for these materials, to underpin their large-scale deployment for applications including the remediation of waters and soils contaminated with metals, radionuclides and organics and also medical applications [42].

## Supplementary Material

Supplementary Tables and Figures

## Data accessibility

All data associated with this publication are available at http://www.pangea.de.

## References

[1] Goya GF Grazu V Ibarra MR 2008 Magnetic nanoparticles for cancer therapy. Curr. Nanosci. 4, 1–16. (10.2174/157341308783591861)

[2] Islam FS Pederick RL Gault AG Adams LK Polya DA Charnock JM Lloyd JR 2005 Interactions between the Fe(III)-reducing bacterium *Geobacter sulfurreducens* and arsenate, and capture of the metalloid by biogenic Fe(II). Appl. Environ. Microbiol. 71, 8642–8648. (10.1128/AEM.71.12.8642-8648.2005) 16332858 PMC1317334

[3] Zeng H Black CT Sandstrom RL Rice PM Murray CB Sun SH 2006 Magnetotransport of magnetite nanoparticle arrays. Phys. Rev. B 73, 020402 (10.1103/PhysRevB.73.020402)

[4] Kanel SR Nepal D Manning B Choi H 2007 Transport of surface-modified iron nanoparticle in porous media and application to arsenic(III) remediation. J. Nanoparticle Res. 9, 725–735. (10.1007/s11051-007-9225-7)

[5] Ehrhardt H Campbell SJ Hofmann M 2002 Structural evolution of ball-milled ZnFe_2_O_4_. J. Alloys Compounds 339, 255–260. (10.1016/S0925-8388(01)02011-4)

[6] Liu C Zou B Rondinone AJ Zhang ZJ 2000 Reverse micelle synthesis and characterization of superparamagnetic MnFe_2_O_4_ spinel ferrite nanocrystallites. J. Phys. Chem. B 104, 1141–1145. (10.1021/jp993552g)

[7] Caccavo F Lonergan DJ Lovley DR Davis M Stolz JF McInerney MJ 1994 *Geobacter sulfurred*ucens sp. nov., a hydrogen- and acetate-oxidizing dissimilatory metal-reducing microorganism. Appl. Environ. Microbiol. 60, 3752–3759. 7527204 10.1128/aem.60.10.3752-3759.1994 PMC201883

[8] Lovley DR Stolz JF Nord GL Phillips EJP 1987 Anaerobic production of magnetite by a dissimilatory iron-reducing microorganism. Nature 330, 252–254. (10.1038/330252a0)

[9] Zhang C Vali H Romanek CS Phelps TJ Liu SV 1998 Formation of single-domain magnetite by a thermophilic bacterium. Am. Mineral. 83, 1409–1418.

[10] Coker VS Pattrick RAD van der Laan G Lloyd JR 2007 Formation of magnetic minerals by non-magnetotactic prokaryotes. In Magnetoreception and magnetosomes in bacteria, vol. 3 (ed. Schüler D), pp. 275–300. Berlin, Germany: Springer.

[11] Lovley DR Chapelle FH 1995 Deep subsurface microbial processes. Rev. Geophys. 33, 365–381. (10.1029/95RG01305)

[12] Lovley DR Holmes DE Nevin KP 2004 Dissimilatory Fe(III) and Mn(IV) reduction. In Advances in microbial physiology, vol. 49, pp. 219–286. New York, NY: Academic Press. 15518832 10.1016/S0065-2911(04)49005-5

[13] Cornell RM Schwertmann U 2003 The iron oxides: structure, properties, reactions, occurences and uses. Weinheim, Germany: Wiley-VCH.

[14] Hansel CM Benner SG Fendorf S 2005 Competing Fe(II)-induced mineralization pathways of ferrihydrite. Environ. Sci. Technol. 39, 7147–7153. (10.1021/es050666z) 16201641

[15] Hansel CM Benner SG Neiss J Dohnalkova A Kukkadapu RK Fendorf S 2003 Secondary mineralization pathways induced by dissimilatory iron reduction of ferrihydrite under advective flow. Geochim. Cosmochim. Acta 67, 2977–2992. (10.1016/S0016-7037(03)00276-X)

[16] Moon JW Rawn C Rondinone A Love L Roh Y Everett SM Lauf RJ Phelps T 2010 Large-scale production of magnetic nanoparticles using bacterial fermentation. J. Ind. Microbiol. Biotechnol. 37, 1023–1031. (10.1007/s10295-010-0749-y) 20544257

[17] Telling ND Coker VS Cutting RS van der Laan G Pearce CI Pattrick RAD Arenholz E Lloyd JR 2009 Remediation of Cr(VI) by biogenic magnetic nanoparticles: an X-ray magnetic circular dichroism study. Appl. Phys. Lett. 95, 163701 (10.1063/1.3249578)

[18] Cutting RS 2010 Optimizing Cr(VI) and Tc(VII) remediation through nanoscale biomineral engineering. Environ. Sci. Technol. 44, 2577–2584. (10.1021/es902119u) 20196588

[19] Coker VS Bell AMT Pearce CI Pattrick RAD van der Laan G Lloyd JR 2008 Time-resolved synchrotron powder X-ray diffraction study of magnetite formation by the Fe(III)-reducing bacterium *Geobacter sulfurreducens*. Am. Mineral. 93, 540–547. (10.2138/am.2008.2467)

[20] Lovley DR Phillips EJP 1986 Availability of ferric iron for microbial reduction in bottom sediments of the fresh-water tidal Potomac River. Appl. Environ. Microbiol. 52, 751–757. 16347168 10.1128/aem.52.4.751-757.1986 PMC239109

[21] Stookey LL 1970 Ferrozine—a new spectrophotometric reagent for iron. Anal. Chem. 42, 779–781. (10.1021/ac60289a016)

[22] Lovley DR Phillips EJP 1988 Novel mode of microbial energy-metabolism—organic-carbon oxidation coupled to dissimilatory reduction of iron or manganese. Appl. Environ. Microbiol. 54, 1472–1480. 16347658 10.1128/aem.54.6.1472-1480.1988 PMC202682

[23] Smith PK 1985 Measurement of protein using bicinchoninic acid. Anal. Biochem. 150, 76–85. (10.1016/0003-2697(85)90442-7) 3843705

[24] Neidhardt FC Ingraham J Schaechter M 1990 Physiology of the bacterial cell: a molecular approach. Sunderland, MA: Sinauer Associates.

[25] Patterson AL 1939 The Scherrer formula for X-ray particle size determination. Phys. Rev. 56, 978–982. (10.1103/PhysRev.56.978)

[26] Pattrick RAD van der Laan G Henderson CMB Kuiper P Dudzik E Vaughan DJ 2002 Cation site occupancy in spinel ferrites studied by X-ray magnetic circular dichroism: developing a method for mineralogists. Eur. J. Mineral. 14, 1095–1102. (10.1127/0935-1221/2002/0014-1095)

[27] van der Laan G Kirkman IW 1992 The 2p absorption spectra of 3d transition metal compounds in tetrahedral and octahedral symmetry. J. Phys. Condens. Matter 4, 4189–4204. (10.1088/0953-8984/4/16/019)

[28] van der Laan G Thole BT 1991 Strong magnetic X-ray dichroism in 2p absorption spectra of 3d transition-metal ions. Phys. Rev. B 43, 13 401–13 411. (10.1103/PhysRevB.43.13401) 9997170

[29] Rancourt DG Ping JY 1991 Voigt-based methods for arbitrary-shape static hyperfine parameter distributions in Mössbauer spectroscopy. Nucl. Instrum. Methods Phys. Res. B 58, 85–97. (10.1016/0168-583X(91)95681-3)

[30] Skougstad MWF Fishman MJ Friedman LC Erdman D Duncan SS 1979 Methods for determination of inorganic substances in water and fluvial sediments. In US Geological Survey techniques of water-resources investigations, book 5, ch. A1. Washington, DC: US Geological Survey.

[31] Lloyd JR Macaskie LE 1996 A novel PhosphorImager-based technique for monitoring the microbial reduction of technetium. Appl. Environ. Microbiol. 62, 578–582. 16535242 10.1128/aem.62.2.578-582.1996 PMC1388780

[32] Esteve-Núñez A Sosnik J Visconti P Lovley DR 2008 Fluorescent properties of c-type cytochromes reveal their potential role as an extracytoplasmic electron sink in *Geobacter sulfurreducens*. Environ. Microbiol. 10, 497–505. (10.1111/j.1462-2920.2007.01470.x) 18093163

[33] Fredrickson JK Zachara JM Kennedy DW Dong H Onstott TC Hinman NW Li S-m 1998 Biogenic iron mineralization accompanying the dissimilatory reduction of hydrous ferric oxide by a groundwater bacterium. Geochim. Cosmochim. Acta 62, 3239–3257. (10.1016/S0016-7037(98)00243-9)

[34] Cutting RS Coker VS Fellowes JW Lloyd JR Vaughan DJ 2009 Mineralogical and morphological constraints on the reduction of Fe(III) minerals by *Geobacter sulfurreducens*. Geochim. Cosmochim. Acta 73, 4004–4022. (10.1016/j.gca.2009.04.009)

[35] Byrne JM Telling ND Coker VS Pattrick RAD van der Laan G Arenholz E Tuna F Lloyd JR 2011 Control of nanoparticle size, reactivity and magnetic properties during the bioproduction of magnetite by *Geobacter sulfurreducens*. Nanotechnology 22, 455709 (10.1088/0957-4484/22/45/455709) 22020365

[36] Momose S Kodama H Uzumaki T Tanaka A 2005 Fine tuning of the sizes of FePt nanoparticles. Jpn. J. Appl. Phys. 44, 1147–1149. (10.1143/JJAP.44.1147)

[37] Nandwana V Elkins KE Poudyal N Chaubey GS Yano K Liu JP 2007 Size and shape control of monodisperse FePt nanoparticles. J. Phys. Chem. C 111, 4185–4189. (10.1021/jp068330e)

[38] Vopsaroiu M Vallejo Fernandez G Thwaites MJ Anguita J Grundy PJ O'Grady K 2005 Deposition of polycrystalline thin films with controlled grain size. J. Phys. D 38, 490–496. (10.1088/0022-3727/38/3/022)

[39] Roh Y Zhang CL Vali H Lauf RJ Zhou J Phelps TJ 2003 Biogeochemical and environmental factors in Fe biomineralization: magnetite and siderite formation. Clays Clay Miner. 51, 83–95. (10.1346/CCMN.2003.510110)

[40] Jacobs IS 1963 Metamagnetism of siderite (FeCO_3_). J. Appl. Phys. 34, 1106–1107. (10.1063/1.1729389)

[41] Crean DE Coker VS van der Laan G Lloyd JR 2012 Engineering biogenic magnetite for sustained Cr(VI) remediation in flow-through systems. Environ. Sci. Technol. 46, 3352–3359. (10.1021/es2037146) 22397548

[42] Lloyd JR Byrne JM Coker VS 2011 Biotechnological synthesis of functional nanomaterials. Curr. Opin. Biotechnol. 22, 509–515. (10.1016/j.copbio.2011.06.008) 21742483

