# Supplementary material for: Scale-up of the production of highly reactive biogenic magnetite nanoparticles using Geobacter sulfurreducens
Source: J R Soc Interface. 2015 Jun 6;12(107):20150240. doi: 10.1098/rsif.2015.0240 (PMC4590511; doi:10.1098/rsif.2015.0240)
Supplement: Supplementary Tables and Figures [file rsif20150240supp1.pdf]

Scale-up of the Production of Highly Reactive Biogenic Magnetite Nanoparticles Using *Geobacter sulfurreducens*

J M Byrne[1,2], H Muhamadali[1,3], V S Coker[1], J Cooper[4], J R Lloyd[1]

1. *School of Earth, Atmospheric and Environmental Sciences, and Williamson Research Centre for Molecular Environmental Science, The University of Manchester, Manchester M13 9PL, UK*
2. *Department of Geosciences, University of Tuebingen, Center for Applied Geoscience Sigwartstrasse 10, 72076 Tuebingen, Germany*
3. *Manchester Institute of Biotechnology, The University of Manchester, Manchester, M1 7DN, UK*
4. *The Centre for Process Innovation, CPI, Wilton Centre, Wilton, Redcar, TS10 4RF*

***corresponding author: jon.lloyd@manchester.ac.uk**

Supplementary information

Key words: Fe(III)-reduction, remediation, bioreactor, nanotechnology, geobiology

Sample	Temp.	Phase	Site	Pop. %	CS (mm/s)	QS (mm/s)	B_{hf} (T)
10 ml	110 K	Magnetite	(A)	44.1	0.44	-0.01	50.8
			[B]	31.9	0.41	-0.04	48.0
		Hematite		24.0	0.44	0.11	53.2
10 L	110 K	Magnetite	(A)	39.3	0.41	-0.01	49.4
			[B]	31.4	0.40	-0.08	46.4
		Siderite		6.3	1.3	2.0*	
		Hematite		23.0	0.47	-0.08	52.0

Supplementary Table S1 – Results of Mössbauer spectroscopy fitting. Temp- - temperature of measurement Pop. – area of relative components; CS – center shift; QS – quadrupole shift (* QS of siderite corresponds to quadrupole splitting); B_{hf} – hyperfine magnetic field. Samples were dominated by magnetite with hematite between 23% and 24% of the total spectral area. Siderite was observed in sample 10 L, 110 K in agreement with XRD data.

Compound	Amount	
Fumaric Acid (40 mM)	4.64	g
**100X NB mix*	*10 ml*	*ml*
***NB Mineral elixir*	*10 ml*	*ml*
****Vitamin mix(DL)*	*15 ml*	*ml*
Calcium Chloride Dihydrate	0.04	g
Magnesium Sulphate Heptahydrate	0.10	g
Sodium Bicarbonate Anhydrous	1.80	g
Sodium Carbonate Monohydrate	0.50	g
*****Sodium Selenate*	*1.00*	*ml*
Sodium Acetate (25 mM)	2.04	g
Water	1000	ml
**<u>100X NB Mix</u>*		
Mono-potassium phosphate	42.0	g
Di-potassium phosphate	22.0	g
Ammonium chloride	20.0	g
Potassium chloride	38.0	g
Sodium chloride	36.0	g
Water	1000	ml
***<u>NB Mineral Elixir</u>*		
Nitrilotriacetic acid	2.140	g
Manganese(II) chloride tetrahydrate	0.100	g
Iron(II) sulphate heptahydrate	0.300	g
Cobalt(II) chloride hexahydrate	0.170	g
Zinc sulphate heptahydrate	0.200	g
Copper chloride dihydrate	0.030	g
Potassium Alum dodecahydrate	0.005	g
Boric acid	0.005	g
Sodium Molybdate	0.090	g
Nickel sulphate hexahydrate	0.110	g
Sodium tungstate dihydrate	0.020	g
Water	1000	ml
****<u>Vitamin Mix</u>*		
Biotin	2.0	mg
Folic acid	2.0	mg
Pyridoxine HCl	10.0	mg
Riboflavin	5.0	mg
Thiamine	5.0	mg
Nicotinic acid	5.0	mg
Pantothenic acid	5.0	mg
B-12	0.1	mg
p-aminobenzoic acid	5.0	mg
Thioctic acid	5.0	mg
Water	1000	ml
*****<u>Sodium Selenate stock solution</u>*		
Sodium selenate	1.90	mg
Water	1000	ml

Supplementary Table S2 – Full list of compounds contained within the modified freshwater medium used to support *Geobacter sulfurreducens* in this study.

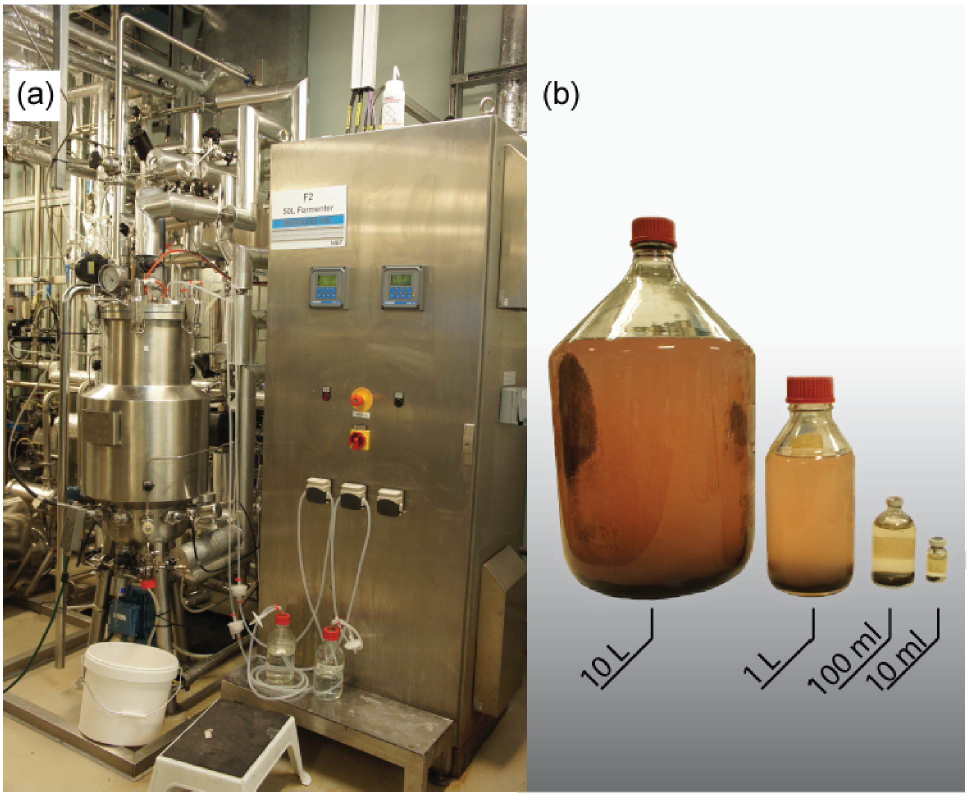

Supplementary Figure S1 – Large-scale production of biogenic magnetite nanoparticles. (a) 50 L bioreactor for batch production of *Geobacter sulfurreducens*. (b) Fe(III)-oxyhydroxide starting material was inoculated with washed cell suspensions and electron donor and an electron shuttle (riboflavin) in bottles of varying volume (10 ml to 10 L).

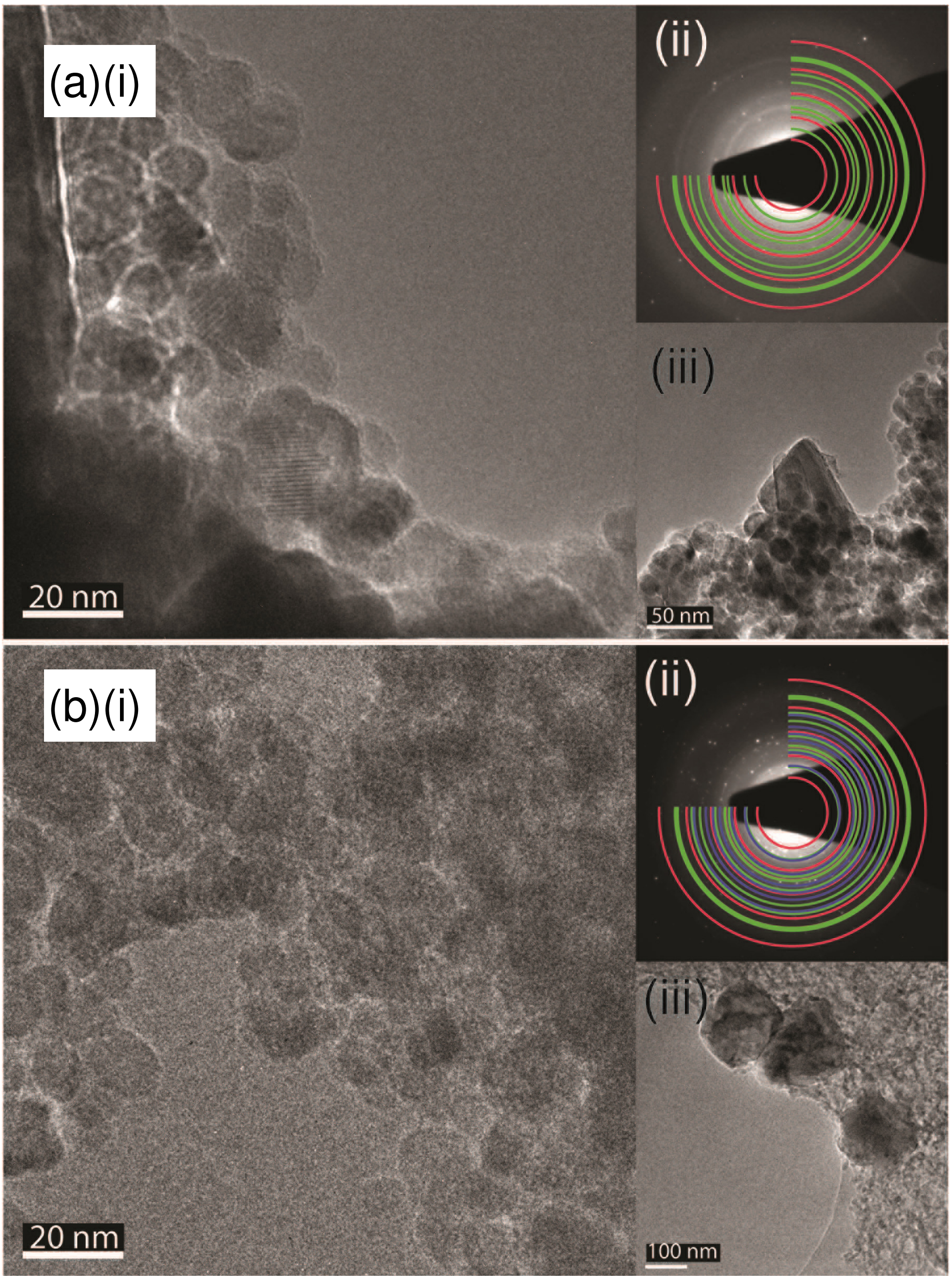

Supplementary Figure S2 – Transmission electron microscopy imaging of end product of Fe(III)-reduction by *Geobacter sulfurreducens*. (a) 10 ml volume vessel, (a-i) image displays spherical particles, most likely magnetite, (a-ii) electron diffraction indicates presence of magnetite (red) and hematite (green), (a-iii) hematite crystal clearly distinguishable from spherical magnetite. (b) 10 L volume vessel sample. (b-i) spherical magnetite of similar size to 10 ml samples, (b-ii) electron diffraction indicates presence of hematite (green), magnetite (red) and siderite (blue); (b-iii) several crystals were observed which were much larger than the relative size of the magnetite that have been attributed to siderite.

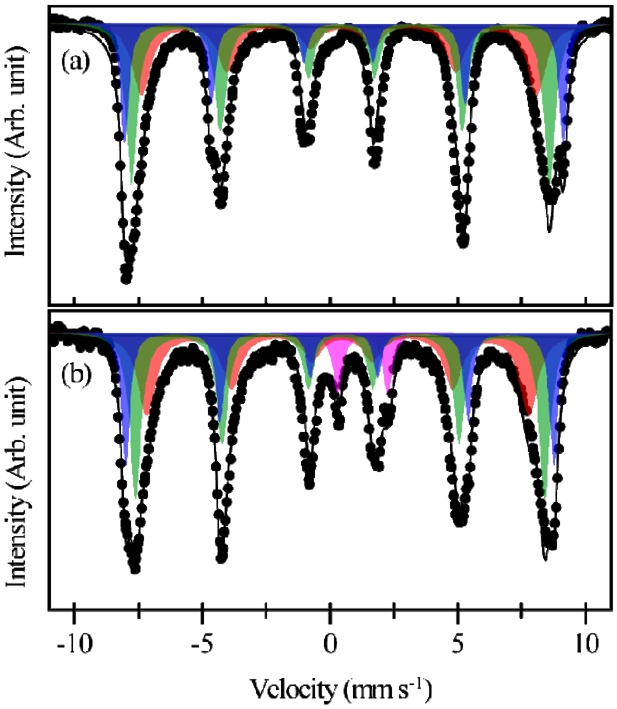

Supplementary Figure S3 – Mössbauer spectroscopy was used to determine the relative abundances of the different iron phases present in the samples. (a) 100 ml experiment, 110 K; (b) 10 L sample, 110 K. Overall fits are represented by grey lines, blue sextet corresponds to hematite, green and red to (A) and [B] sites of magnetite respectively and magenta to siderite.
